# Global and Regional Sex-Related Differences, Asymmetry, and Peak Age of Brain Myelination in Healthy Adults

**DOI:** 10.3390/jcm13237065

**Published:** 2024-11-22

**Authors:** Marina Y. Khodanovich, Mikhail V. Svetlik, Anna V. Naumova, Anna V. Usova, Valentina Y. Pashkevich, Marina V. Moshkina, Maria M. Shadrina, Daria A. Kamaeva, Victoria B. Obukhovskaya, Nadezhda G. Kataeva, Anastasia Y. Levina, Yana A. Tumentceva, Vasily L. Yarnykh

**Affiliations:** 1 Laboratory of Neurobiology, Research Institute of Biology and Biophysics, Tomsk State University, 36 Lenina Ave., Tomsk 634050, Russia; 2 Department of Radiology, School of Medicine, South Lake Union Campus, University of Washington, 850 Republican St., Seattle, WA 98109, USA; 3 Cancer Research Institute, Branch of the Tomsk National Research Medical Center of the Russian Academy of Sciences, 5 Kooperativny St., Tomsk 634009, Russia; 4 Laboratory of Molecular Genetics and Biochemistry, Mental Health Research Institute, Tomsk National Research Medical Center of the Russian Academy of Sciences, 4 Aleutskaya St., Tomsk 634014, Russia; 5 Department of Fundamental Psychology and Behavioral Medicine, Siberian State Medical University, 2 Moskovskiy Trakt, Tomsk 634050, Russia; 6 Department of Neurology and Neurosurgery, Siberian State Medical University, 2 Moskovskiy Trakt, Tomsk 634050, Russia; 7 Medica Diagnostic and Treatment Center, 86 Sovetskaya St., Tomsk 634510, Russia

**Keywords:** sex, gender, myelin, quantitative MRI, neuroimaging, macromolecular fraction mapping, magnetization transfer, MPF, white matter, asymmetry, interhemispheric differences, age, body mass index, BMI

## Abstract

**Background:** The fundamental question of normal brain myelination in human is still poorly understood. **Methods**: Age-dependent global, regional, and interhemispheric sex-related differences in brain myelination of 42 (19 men, 23 women) healthy adults (19–67 years) were explored using the MRI method of fast macromolecular fraction (MPF) mapping. **Results**: Higher brain myelination in males compared to females was found in global white matter (WM), most WM tracts, juxtacortical WM regions, and putamen. The largest differences between men and women, exceeding 4%, were observed bilaterally in the frontal juxtacortical WM; angular, inferior occipital, and cuneus WM; external capsule; and inferior and superior fronto-orbital fasciculi. The majority of hemispheric differences in MPF were common to men and women. Sex-specific interhemispheric differences were found in juxtacortical WM; men more often had left-sided asymmetry, while women had right-sided asymmetry. Most regions of deep gray matter (GM), juxtacortical WM, and WM tracts (except for projection pathways) showed a later peak age of myelination in women compared to men, with a difference of 3.5 years on average. Body mass index (BMI) was associated with higher MPF and later peak age of myelination independent of age and sex. **Conclusions**: MPF mapping showed high sensitivity to assess sex-related differences in normal brain myelination, providing the basis for using this method in clinics.

## 1. Introduction

A detailed understanding of the anatomical and morphological differences between the normal male and female brain is important from several points of view: First, there is sexual dimorphism in a variety of neurological and psychiatric diseases. Knowledge of sex-related morphological and functional differences could help explain why some psychiatric and neurological disorders are predominant in men or women. Second, in any study of myelin-related disorders, sex-related differences should be considered when selecting control groups of healthy volunteers. Third, a detailed understanding of sex-related brain morphological differences will help to understand the physiological basis of the psychological and behavioral characteristics of thinking, memory, and emotions in men and women.

It is known that the total brain volume is larger in men compared to women [1,2,3,4], as well as the absolute volume of the WM compartment. Most studies have used only volumetric methods to examine sex-related differences in white matter (WM) and gray matter (GM), but not differences in myelin density. Surprisingly, we found relatively few studies focused on the differences between adult men and women in myelin by quantitative MRI [3,4,5,6,7,8,9,10,11,12,13,14,15,16,17,18,19,20,21,22,23,24,25,26,27], especially both sex and age differences [24,26,28]. The influence of body mass index (BMI), potentially related to myelin production and metabolism [29,30,31], is also rarely taken into account in such studies.

Among the quantitative myelin-sensitive magnetic resonance imaging (MRI) methods, diffusion tensor imaging (DTI) has been the most common approach to study sex-related WM differences. DTI studies more often reported greater fractional anisotropy (FA) in males compared to females in various WM tracts, juxtacortical WM, and deep GM [4,5,11,12,13,15,17,21,22,23,24,27]. There is no agreement across these studies regarding the brain structures for which sex-related differences were found. Several studies did not find sex-related differences in DTI parameters [9,10] or found opposite sex-related differences in various brain structures [5,15,24,27]. It is known that the main limitation of DTI for myelin assessment is the sensitivity of FA to the direction of diffusion in areas containing fibers in different directions [32]. This limitation, as well as significant differences in segmentation and image analysis methods, could explain such heterogeneous results. A more myelin-specific method, myelin water fraction (MWF) mapping, is less commonly used to study sex-related differences [16,17,18,19,20,33]. Some studies found that males have higher MWF in the CC, temporal juxtacortical WM, arcuate, uncinate, and inferior longitudinal fasciculi [16,17]. There was a study showing a decrease in MWF with age in women [20], while other studies showed no sex-related differences [18,19,33]. There are too few studies yet to draw any conclusions.

A recently developed myelin-specific macromolecular proton fraction (MPF) mapping method was used in this study to investigate sex-specific differences and sex-by-age interactions in brain myelination. The method is based on the magnetization transfer (MT) effect but differs significantly from a better-known semi-quantitative MT ratio (MTR) index, which has lower specificity and sensitivity for myelin due to its dependence on longitudinal relaxation [34,35] and, therefore, paramagnetic effects. An alternative and more comprehensive way to characterize the MT effect is based on quantitative mapping of the fundamental parameters of the two-pool MT model [36]. One of the parameters of the two-pool model, macromolecular proton fraction (MPF), has attracted the greatest interest as a myelin biomarker (reviewed in [37]). The fast single-point MPF mapping method [38,39] allows reconstruction of MPF maps in isolation from other parameters of the two-pool model and overcomes the limitations of most MT-based methods related to data acquisition time and sensitivity to noise. The MPF mapping method is independent of magnetic field strength [40], myelinated fiber orientation [32], and iron deposition [41], shows high reproducibility [42,43,44], and is easy to use on routine clinical equipment without modification of the original pulse sequences [44,45]. Animal models have demonstrated a strong correlation between MPF and myelin histology in normal animal brain [34,42] and animal models of cuprizone-induced demyelination and remyelination [42,46], ischemic stroke [47,48] and neonatal development [49]. Clinical applications of fast MPF mapping in studies of multiple sclerosis (MS) [35,41], mild traumatic brain injury [50], schizophrenia [44], and normal brain development in fetuses [51,52], children [53,54], adolescents [55], and aging [45] confirmed the feasibility of using this method in humans.

While MPF mapping becomes a popular myelin imaging technique, the studies of the age and sex dependence of normal brain myelination based on this method are extremely limited. The published reports investigated myelination during relatively short periods of time, including childhood [53,54], adolescence [55], and prenatal development [51,52].

Our recent longitudinal study with a 7-year interval between scans [45] demonstrated a significant age-related decline in brain myelination in healthy adults, but sex-by-age interactions were not investigated. The present study fills this gap to examine specific sex-related differences, asymmetry in myelination in adults, and the interactions between age and sex, taking into account the influence of BMI.

## 2. Materials and Methods

### 2.1. Study Participants

Forty-one healthy participants were recruited between September 2022 and June 2023. The inclusion criteria were as follows: age from 18 to 70 years, the absence of the history of traumatic brain injury, and the absence of any diagnosed neurologic or psychiatric condition. The exclusion criteria were as follows: pregnancy, symptoms of acute infectious and somatic diseases, inability to tolerate the MRI procedure, contraindications to MRI, and self-withdrawal from the study. All participants were right-handed. Written, informed consent was obtained from all participants. The study design was approved by the local Ethical Committee of the Mental Health Research Institute (protocol №15/8.2022 dated 25 August 2022) and Bioethics Committee of Tomsk State University (№12/06.2022 dated 6 June 2022) following the guidelines of the Declaration of Helsinki.

The demographic characteristics of participants are shown in Table 1. Men and women did not differ significantly in age, BMI, and education.

### 2.2. MRI Data Acquisition

MRI data were collected using a 1.5 T clinical scanner Magnetom Essenza (Siemens, Erlangen, Germany). The fast MPF mapping protocol [44] included three 3D spoiled gradient-echo pulse sequences with the following acquisition parameters:

- Magnetization transfer weighted (MTw): TR = 20 ms, echo time (TE) = 4.76 ms, flip angle (FA) = 8°, acquisition time = 5 min 40 s;
- T1-weighted (T1w): TR =16 ms, TE = 4.76 ms, FA =18°, acquisition time = 4 min 32 s;
- Proton density weighted (PDw): TR= 16 ms, TE = 4.76 ms, FA= 3°, acquisition time = 4 min 32 s.
- In addition, the following sequences were included into the scanning protocol:
- 3D FLAIR: TR = 5000 ms, TE = 390 ms, TI = 1800 ms;
- 3D T1w: TR = 16 ms, TE = 4.76 ms;
- 3D T2w: TR = 3000 ms, TE = 335 ms.

All scans were acquired in the sagittal plane with a voxel size = 1.25 × 1.25 × 1.25 mm^3^, matrix 192 × 192 × 160, FOV = 240 × 240 × 200 mm^3^, and single signal averaging.

The total scanning time was about 35 min per person.

### 2.3. Image Processing

**Figure 1:**
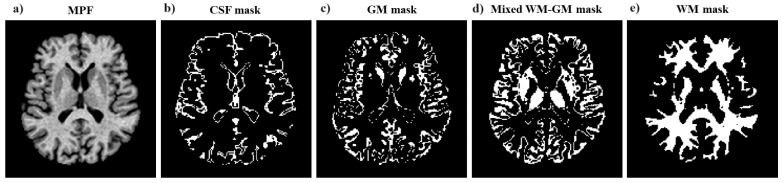
Example MPF map (**a**) and corresponding masks used for global measurements: cerebrospinal fluid (CSF) (**b**), GM (**c**), WM (**e**), and mixed WM–GM (**d**).

MPF maps were reconstructed using a single-point algorithm with a synthetic reference image [38,56] and the previously developed software in the C++ language (available at https://www.macromolecularmri.org/, assessed on 1 June 2024). Example MPF maps are shown in Figure 1a and Supplementary Figure S1.

Global WM and GM measurements on skull-stripped MPF maps. A mask for skull stripping was obtained by applying the BET algorithm to the PD-weighted images in the MRIcro application [57]. The mask was then converted to a binary image using the Threshold function in ImageJ (Fiji) [58] and applied to the MPF maps to remove extracerebral tissue. Automatic global segmentation of MPF maps was performed using the FSL [59] package. The masks of WM, GM, mixed WM–GM, and mixed GM with cerebrospinal fluid (CSF) were obtained as detailed earlier [35,44]. Bias field correction was not used since MPF maps are not affected by the coil reception profile and have negligible sensitivity to B1 field inhomogeneity (particularly at 1.5 T [43]). Masks were used to measure the mean MPF values and volumes of WM and mixed WM–GM compartments. To avoid errors associated with differences in male and female brain size, the relative volumes of the compartments and the ratio of the WM/GM volume was also calculated. The measurements were carried out using ITK-SNAP software (version 3.6.0) [60].

**Figure 2:**
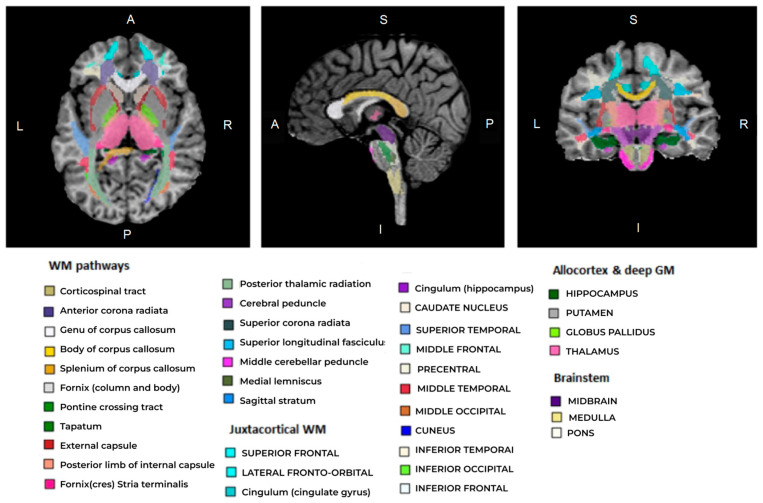
An example of an individual MPF map segmentation obtained by registration of T1 Eve template [63] to MPF map. Slices are shown in axial, sagittal, and coronal projections. Different colors indicate regional segmentation of separate brain structures of juxtacortical WM, WM pathways, allocortex, deep GM, and brainstem.

Regional WM and GM segmentation was performed using Advanced Normalization Tools (ANTs), version v.2.4.4, [61,62] and Eve anatomical atlas [63], as described in [45]. T1 template image of Eve atlas was registered to individual MPF maps using antsRegistrationSyNQuick algorithm. The obtained deformation field was applied to Type-III Eve atlas segmentation [63] to register the template atlas labels to individual MPF maps (Figure 2).

The measurements on MPF maps were obtained for 118 GM and WM structures (including measurements in the right and left hemispheres) using ITK-snap software. The list of structures included the following:

1. Juxtacortical (superficial) WM: superior, middle, and inferior frontal; lateral and middle fronto-orbital; precentral; postcentral; superior parietal; angular; pre-cuneus; cuneus; lingual; fusiform; superior, inferior, and middle occipital; superior, inferior, and middle temporal; supramarginal; rectus; and cingulum (cingular and hippocampal parts);
2. WM pathways and fasciculi: corticospinal tract (CST); anterior, superior, and posterior corona radiata (CR); genu, body, and splenium of corpus callosum (CC); anterior limb, posterior limb, and retrolenticular part of internal capsule (IC); inferior, superior, and middle cerebellar peduncles (CP); cerebral peduncles; posterior thalamic radiation; fornix (FX) (stria terminalis, column, and body); medial lemniscus; superior longitudinal (SL) fasciculus; superior (SFO) and inferior fronto-occipital (IFO) fasciculi; uncinate fasciculus; sagittal stratum; external capsule; pontine crossing tract; tapetum;
3. Allocortical and deep GM structures: amygdala; hippocampus; entorhinal area; thalamus; caudate nucleus; putamen; globus pallidus;
4. Brainstem structures: medulla pons; midbrain.

The measurements for left and right hemispheres for the midbrain, pons, and medulla were averaged. Other brain structures were analyzed separately for the left or right hemisphere.

### 2.4. Statistical Analysis

Statistical analysis was performed using Statistica 10.0 software. Differences in MPF between female and male participants, sex-related age influence, and sex-related interhemispheric differences for each brain structure were analyzed using the general linear models and nonlinear estimation modules. The homogeneity of variances for male and female sub-samples were assessed for all variables using Levene’s test. The model for global measurements included intergroup factor “sex” (2 levels), repeated measures factor “matter” (2 levels—WM, mixed WM–GM), and the factor “age” as a covariate. The model for separate brain structures included intergroup factor “sex” (2 levels), repeated measures factor “hemisphere” (2 levels), and the factors “age” and body mass index (“BMI”) as covariates. Post-hoc Fisher LSD tests adjusted to covariates were performed to clarify sex-related and interhemispheric differences. The age and BMI effects, as well as interactions of “sex” with “age” and BMI, was also analyzed for global compartments and for each brain structure. Regression analysis was performed for each of the brain structures separately for men and women. The relationship between MPF and age for the total sample and separately for men and women was examined by fitting linear and quadratic functions. If the fitting of the quadratic function was significant, peak age was calculated from the coefficients of the quadratic equation. Additionally, the impact of BMI on sex-dependent MPF differences and the peak age of myelination was investigated. Age-controlled partial correlations between BMI and MPF measurements were calculated for the total sample and for men and women separately. To assess the effect of BMI on peak age of myelination, participants were divided into two groups: BMInorm group (9 men, 10 women) with BMI ≤ 25, and BMIex group (13 men, 10 women) with BMI > 25. Peak age was estimated using a quadratic model for each group and for men and women within each group. A one-way ANOVA was used to assess between-group differences in age, BMI, and education. For analyses with degrees of freedom > 2, *p*-values were adjusted using the Benjamini–Hochberg false discovery rate (FDR) correction procedure to prevent false positives from multiple comparisons. In all analyses, the differences were considered statistically significant at *p* < 0.05.

## 3. Results

### 3.1. Global Age-Modified Sex-Related Differences in Brain Myelination

Example MPF maps, T1w, T2w, and T2-FLAIR images of male and female brain are shown in Supplementary Figure S1. The larger volume of the male brain compared to the female brain is clearly visible. There are no visible sex-related differences in MPF maps.

**Figure 3:**
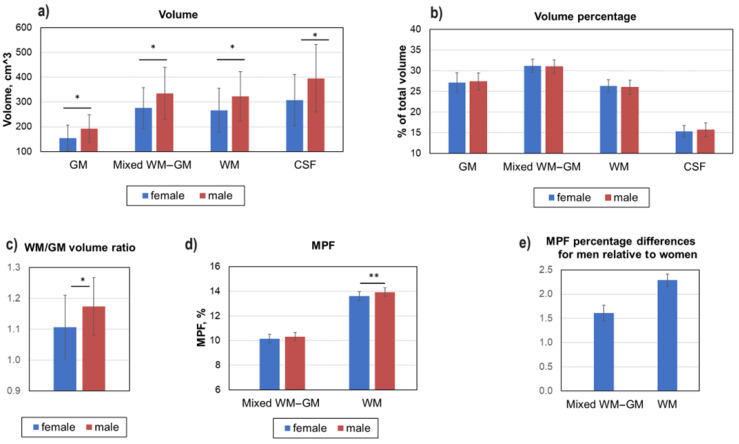
Sex-related global differences in brain myelination and volumes for WM, GM, mixed WM–GM, and CSF compartments. (**a**) Absolute volume differences in GM, WM, mixed WM–GM, and CSF. (**b**) Percentage differences in GM, WM, mixed WM–GM, and CSF. (**c**) Ratio of WM volume to GM volume. (**d**) Absolute MPF differences in global GM, WM, and mixed WM–GM. (**e**) Percentage MPF differences in global GM, WM, and mixed WM–GM. Error bars denote standard deviation. Significant differences: *—*p* < 0.05, **—*p* < 0.01.

Figure 3 demonstrates age-adjusted global changes in MPF and volumes of WM, GM, CSF, and mixed WM–GM compartments. As expected, the absolute volume of GM, mixed WM–GM, and WM compartments was significantly higher in the male compared to female brain (Figure 3a). No sex-based differences in the relative volumes of all compartments were found (Figure 3b). However, the ratio of white matter volume to gray matter volume is significantly higher in the male brain (Figure 3c).

Significantly higher MPF on global WM was observed in men compared to women (Figure 3d). The MPF of WM in men is 2.29 ± 0.13% higher than that of women (Figure 3e), while MPF in global GM and mixed WM–GM differ by only 0.10 ± 0.16% and 1.61 ± 0/16%, respectively, and are not statistically significant.

### 3.2. Sex-Related Differences in Separate WM and GM Structures

**Figure 4:**
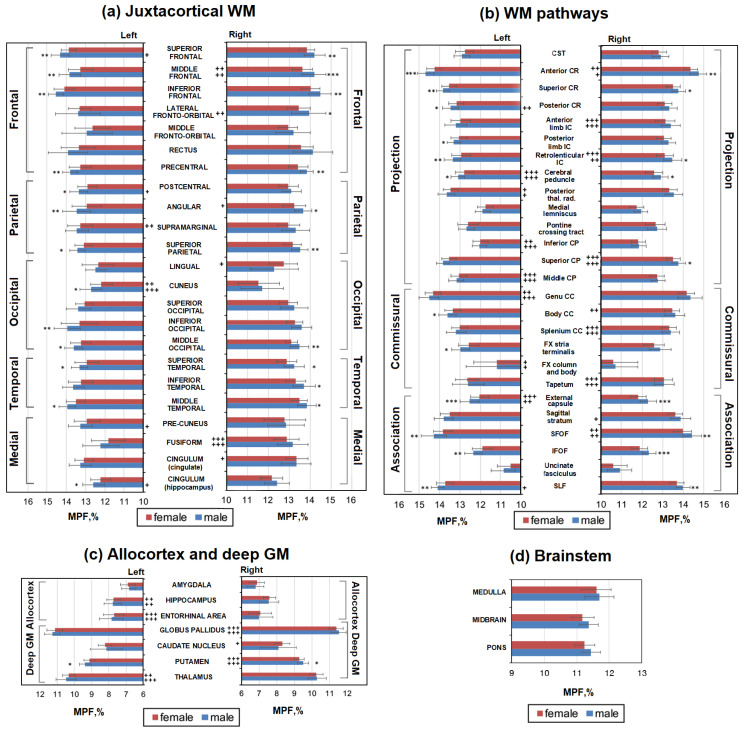
Sex-related differences between MPF measurements in men and women for the separate brain regions: (**a**) juxtacortical WM of left and right hemispheres, (**b**) WM pathways in left and right hemispheres, (**c**) allocortex and deep GM of left and right hemispheres, (**d**) brainstem. Significant differences between men and women: *—*p* < 0.05, **—*p* < 0.01, ***—*p* < 0.001. Significant differences between left and right hemispheres: +—*p* < 0.05, ++—*p* < 0.01, +++—*p* < 0.001. The significance of the differences is marked on the side of the hemisphere in which the MPF is larger. Error bars correspond to SD.

**Figure 5:**
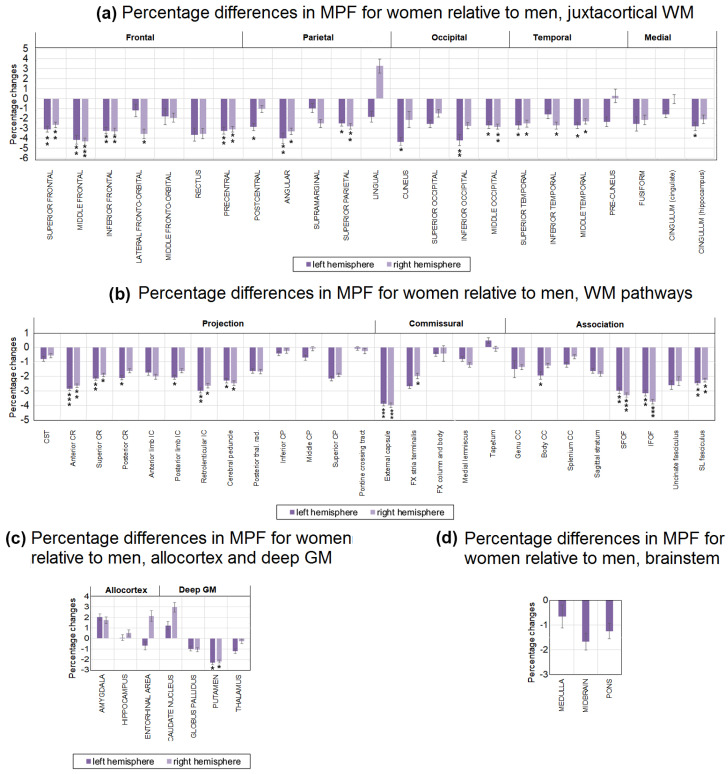
Sex-related percentage differences in average MPF measurements for women compared with men in the separate brain regions: (**a**) juxtacortical WM of left and right hemispheres, (**b**) WM pathways of left and right hemispheres, (**c**) allocortex and deep GM of left and right hemispheres, (**d**) brainstem. Significant differences between men and women: *—*p* < 0.05, **—*p* < 0.01, ***—*p* < 0.001. Error bars correspond to SD.

**Figure 6:**
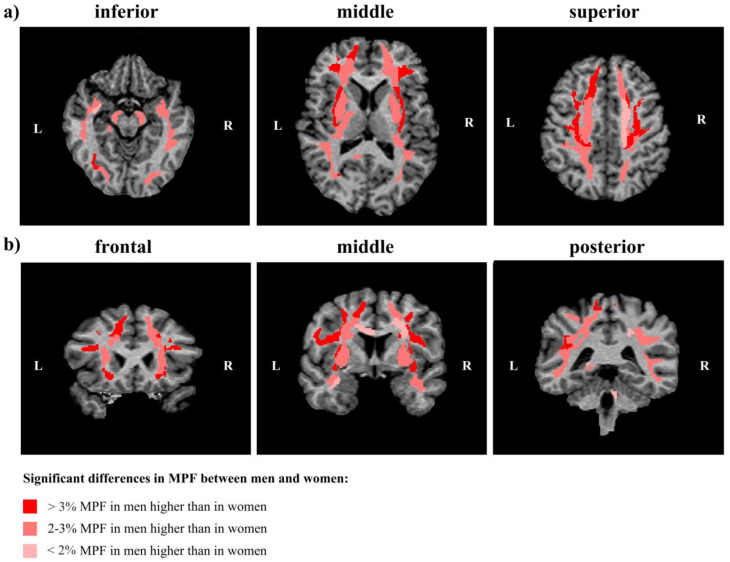
Regions of significant sex-related differences between MPF measurements in women and men in representative cross-sections of a 3D MPF map at different levels of axial (**a**) and coronal (**b**) projections of an individual MPF map. Regions of significantly higher MPF in men are marked by red (>3%) and pink (<3%) colors. L—left hemisphere, R—right hemisphere.

The absolute and percentage sex-related differences in MPF for separate brain structures are shown in Figure 4 and Figure 5. Significant differences between men and women already take into account adjustment for age as a covariate. As seen from Figure 5 and Figure 6, MPF is higher in men relative to women in most WM pathways, juxtacortical WM regions, and the brainstem. Significantly higher MPF values in men compared to women were found for many structures of juxtacortical WM, subcortical WM pathways, and one GM structure (Figure 4a,b).

In the frontal lobe, the MPF was significantly higher in men compared with women in the left and right superior, middle, inferior frontal, precentral, and the right fronto-orbital juxtacortical WM. The percentage differences between men and women were greatest in the frontal lobe, exceeding 4% for the middle frontal WM and 3% for precentral, superior, and inferior frontal WM. Higher MPF values in men compared with women were also found for juxtacortical WM of the parietal, occipital, and temporal lobes. In the parietal lobe, differences were significant for the left and right superior parietal, angular, and left postcentral WM. In the occipital lobe, differences were significant for the left and right middle occipital, left inferior occipital, and cuneus WM. In the temporal lobe, differences were significant for the left and right superior temporal, middle temporal, and right inferior temporal WM. Sex-related percentage differences in these regions did not exceed 3%, except for the angular, inferior occipital, and cuneus WM, where the differences reached 4%.

A significant MPF decrease in women compared with men was found for the majority of the investigated WM pathways including CR (left and right anterior, superior, and left posterior parts), external capsule, IC (retrolenticular part and left posterior limb), CC (left body of CC), cerebral peduncles, CP (right superior part), FX (left stria terminalis), SFO, SL, and IFO fasciculi. Among the WM tracts (Figure 6b), the external capsule, IFO, and SFO fasciculi achieved the largest sex-related difference of more than 3%. Allocortex, subcortical GM, and brainstem did not show significant differences between men and women, except for the bilateral putamen, where MPF was 2% higher in men compared to women (Figure 5c,d).

Figure 6 summarizes our findings and show regions and magnitude of significant sex-related differences in MPF in representative cross-sections of a 3D MPF map.

### 3.3. Interhemispheric Differences in Brain Myelination of Men and Women

**Figure 7:**
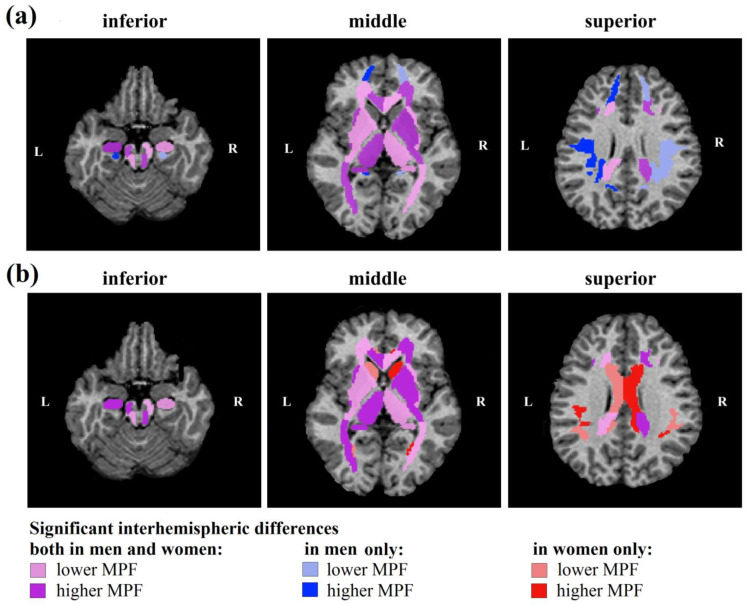
Regions of significant interhemispheric differences in MPF measurements for men (**a**) and women (**b**) at different levels of axial projections of an individual MPF map. Symmetrical areas of the right (R) and left (L) hemispheres with significantly higher and lower MPF values are marked by a more saturated vs. paler tone of red for women, blue for men, and purple for similar interhemispheric differences for men and women.

Figure 4 and Figure 7 show age-adjusted interhemispheric differences in MPF measurements, which were analyzed separately for men and women. For all WM pathways, most deep gray matter structures, and several regions of the juxtacortical white matter regions, similar differences between the left and right hemispheres were found in men and women.

Regardless of sex, MPF of WM pathways (Figure 4b) in the right hemisphere was higher for the splenium of CC, IC (anterior and retrolenticular parts), anterior CR, superior CP, SFO fasciculus, and tapetum, while MPF in the left hemisphere was higher for the genu of the CC, external capsule, posterior thalamic radiation, middle and inferior CP, cerebral peduncles, and FX (column and body). Male-specific interhemispheric differences were found for posterior CR and sagittal stratum: MPF is higher in the right sagittal stratum and left posterior CR compared to symmetrical regions of contralateral hemisphere. Female-specific differences were found only for the body of the CC that showed higher MPF in the right hemisphere.

Similar MPF asymmetry in men and women was also found for most allocortical regions and deep GM (Figure 4c). Regardless of sex, the MPF is higher in the right globus pallidus and putamen and left hippocampus, thalamus, and entorhinal area compared to the contralateral side. In the caudate nucleus, interhemispheric differences were found only for women; MPF is higher in the right hemisphere.

Among juxtacortical WM regions, similar significant asymmetry for men and women was observed only for the middle frontal, fusiform, and cuneus WM; MPF was higher in right middle frontal, right fusiform, and left cuneus WM regardless of sex. Male-specific interhemispheric differences were found for five juxtacortical regions: MPF is higher in the left superior frontal, postcentral, cingulum (hippocampal part), precuneus, and right lateral fronto-orbital WM compared to the contralateral side. In four juxtacortical regions, significant interhemispheric differences were observed in women only; MPF is higher in the left supramarginal and right lingual, cingulum (cingulate part), and angular WM compared to the contralateral side.

### 3.4. Age-Related Differences in Brain Myelination of Men and Women

Table 2 presents the results of the study of sex-related influence of age on global MPF and volumetric measurements. A significant effect of age as a covariate, as well as the interaction of age and sex, was found for the absolute volumes of all compartments excluding CSF volume. As expected, total brain volume and absolute volumes of WM, GM, CSF, and mixed WM–GM compartments significantly decrease with age. These age-related changes are significant for the total sample (r = −0.33 ÷ −0.43) but are more pronounced for men (r = −0.55 ÷ −0.76), in whom the absolute volumes of all compartments, including CSF, decrease. In contrast, percentage CSF volume increases with age; the changes are significant for the total sample (r = 0.44), but correlation in men is stronger (r = 0.71). In women, unlike men, the percentage volume of mixed WM–GM decreases with age (r = −0.64, *p* < 0.01).

For MPF global measurements, a significant effect of age was found only for mixed WM–GM and a significant interaction of age and sex was found only for GM. Regression analysis showed that the quadratic model better fits the dependence of MPF on age. For all compartments, significant quadratic regression models were fit both for the total sample and for men and women separately. The peak age calculated from a quadratic model for all compartments is higher in women than in men. Linear Pearson’s correlation is significant for GM and mixed WM–GM only for women and is positive.

**Figure 8:**
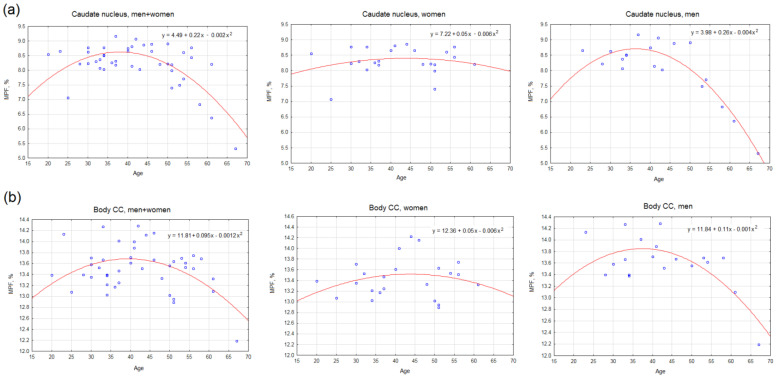
Examples of the best-fitting quadratic curves of age regressed on MPF in the right caudate nucleus (**a**) and the right body of the CC (**b**) for total sample (left), men (center), and women (right). Each scatterplot includes the quadratic regression equation. All shown regression equations are significant (*p* < 0.05). The peak age calculated from the regression equations: (**a**) the caudate nucleus—38 years for the total sample, 36.4 years for men, and 43.8 years for women; (**b**) the body of the CC—39.3 years for the total sample, 37.6 years for men, and 43.5 years for women.

Figure 8, Table 3, and Supplementary Table S1 demonstrate the effect of age on MPF measurements in separate brain structures. Figure 8 shows the examples of the best-fitting quadratic curves for two brain structures. It is evident that the regression curve of the dependence of MPF on age for women has a flatter shape than for men, and its vertex is shifted towards older ages and higher MPF. Such differences in the shape of the curve for men and women were observed for most investigated brain structures for which it was possible to fit a significant quadratic model.

The results of the linear and nonlinear regression analyses, as well as the significance of age as a covariate and the interaction of age and sex, for the full list of brain structures are presented in Supplementary Table S1. Table 3 summarizes our findings and presents the results of quadratic model fitting considering functionality and localization of brain structures. Of the 117 structures studied, a significant quadratic model could be fitted for 116 structures for men and only for 90 structures for women. No significant models could be constructed for temporal WM in women. The peak age calculated from the regression equations for all groups of structures, except for projection WM pathways, is higher in women than in men. The difference in peak age between men and women is 11.4 years for allocortex and deep GM (*p* < 0.05), 3.5 years for juxtacortical WM (*p* < 0.05), 0.6 years for WM pathways (n.s.), 1.2 years for the brainstem (n.s.), and 3.5 ± 9.2 years for all investigated brain structures on average (*p* < 0.01). Interestingly, for association WM pathways, the average peak age is higher in women, while for projection WM pathways, it is higher in men.

### 3.5. Effect of BMI on Brain Myelination of Men and Women

BMI did not differ between the male and female sub-samples (Table 1). Thirteen women and ten men from the total sample who were included in the BMTex group had a BMI > 25 (28.60 + 2.99 on average), while two men and two women from the total sample had a BMI > 30.

Table 4 presents the effect of BMI on MPF and volumetric measurements in men and women within the linear model. Because a weak but significant correlation (r = 0.34, *p* < 0.05) was found between BMI and age, associations of BMI with MFF and volumetric measurements were examined using age-controlled partial correlations. A significant age-adjusted effect of BMI and positive correlations were found for MPF measurements in global WM and WM–GM compartments, while the BMI-by-sex interactions were non-significant. An age-adjusted positive correlation between BMI and MPF in mixed WM–GM compartment was also fount for women. Among all volumetric measurements, only CSF percentage volume was influenced by BMI. Specifically, a significant interaction between these parameters and a negative correlation in women were found.

Table 5 demonstrates the effect of BMI on the peak age of myelination within the quadratic model. A similar trend is observed for both the overall sample and for men and women separately; the peak age of myelination is larger in the overweight participant (BMIex) group compared to the participants with lower BMI (BMInorm). For WM and WM–GM compartments, this difference is 4.5 and 2.6 years for the total sample, 5.2 and 1.4 years for women, and 5.4 and 4.5 years for men, respectively.

## 4. Discussion

This study investigated sex-related differences in WM and GM myelin density on a relatively large sample of adults using quantitative MPF mapping for the first time.

We found significantly greater MPF values in men compared to women in global WM compartment, many juxtacortical WM regions, underlying WM pathways, and one deep GM structure (putamen). The largest differences between men and women, exceeding 4%, were observed bilaterally in the frontal juxtacortical WM, angular, inferior occipital, and cuneus WM; external capsule, IFO, and SFO fasciculi. Our results are in good agreement with a recent study by Corrigan et al. [55] on a large sample of adolescents with the use of MPF mapping, which showed higher MPF in males compared to females across all cortical lobes and in bilateral subcortical regions. It should also be pointed out that our recent longitudinal study [45] of age-related MPF changes found no significant differences between men and women due to a limited sample size.

Sex-related myelin differences in adults were extensively studied using DTI [3,4,5,6,7,8,9,10,11,12,13,14,15,21,22,23,24,25,26,27] and MWF [16,17,18,19,20] quantitative MRI. Most of these studies consistently showed higher myelin density in men compared to women, both in global measurements and in specific brain regions, which is consistent with our results. Greater FA in men was found in the frontal [5,23,24], temporal [5,23,24], parietal [5], and medial [5] juxtacortical WM; CC [11,12,16,17,21,22,27]; cerebellar WM [15]; FX [20]; cingulum [11,26]; internal capsule [13]; CR [13]; posterior thalamic radiation [13]; sagittal stratum [13]; deep GM [4,5]; midbrain [4]; and uncinate, arcuate, superior, and inferior longitudinal fasciculi [5,13,16]. Several studies reported higher FA in men for some regions and in women for others [5,15,24,27]. A number of studies did not find sex-related differences in DTI parameters [9,10] or MWF [18,19,33].

We found both general and sex-specific interhemispheric differences in myelin density for WM and subcortical GM structures. Most hemispheric differences are common to men and women, especially for WM pathways and deep GM. In both men and women, rightward asymmetry in MPF was found for the splenium of CC; anterior and retrolenticular IC; anterior CR; superior CP; SFO fasciculus; tapetum, middle, and inferior CP; cerebral peduncles; column and body of FX; middle frontal and fusiform WM; globus pallidus; and putamen. Meanwhile, leftward asymmetry in MPF was observed in the genu of the CC, external capsule, posterior thalamic radiation, cuneus WM, hippocampus, thalamus, and entorhinal area. It should be noted that the above findings regarding the more myelinated side plausibly agree with the results of a previous study [45] in which interhemispheric differences were identified for juxtacortical WM.

Interestingly, our results suggest that sex-related interhemispheric differences are more pronounced in the juxtacortical WM, and men are more likely to have left-sided asymmetry, while women are more likely to have right-sided asymmetry. Thus, only men show left-sided asymmetry in the superior frontal, postcentral, hippocampal cingulum WM, and posterior CR and right-sided asymmetry in the lateral fronto-orbital WM and sagittal stratum. Female-specific interhemispheric differences are limited to leftward asymmetry in supramarginal WM and rightward asymmetry in the body of the CC, lingual, cingulate cingulum, angular WM, and caudate nucleus.

There are relatively few studies that have examined hemispheric differences in the context of myelin-related parameters. Most have not examined sex-related general and specific asymmetries. Kang et al. [7] found significant left-hemisphere asymmetries in FA in the whole hemisphere, as well as in the corona radiata and juxtacortical WM regions, except for the frontal lobe and limbic cortex. The most prominent pericortical asymmetries in FA were observed in the insula and peri-Sylvian language regions. Kubicki et al. [64] reported leftward asymmetry in the FA of the uncinate fasciculus. Park et al. [65] found leftward FA asymmetry in the anterior part of the CC, cingulum bundle, optic radiation, and superior cerebellar peduncle and rightward FA asymmetry in the anterior limb of the IC, prefrontal superficial regions, uncinate fasciculus, and SLF. As in the latter study, our results suggest a more complex pattern of interhemispheric differences than just a higher myelination in the left hemisphere. However, the literature data are still too sparse to draw clear conclusions about the persistence of these features in the general population. Associations of interhemispheric differences in myelin density with handedness and behavioral traits also remain open questions.

A few studies compared hemispheric differences in myelin density between men and women using quantitative MRI. Steinmann et al. [28] found no significant asymmetry of the SLF and SFOF in healthy males compared to healthy females. Huster et al. [26] found a higher FA in the left mid cingulum bundle, and this asymmetry was more prominent in males compared to females. In contrast with this study, we found right-side asymmetry of cingulate cingulum only in women but not in men. Szeszko et al. [24] reported that females, unlike males, exhibit a leftward frontal asymmetry in FA, which correlates with a better comprehension of verbal constructions and memory functioning in women. Our study, in contrast, shows greater frontal asymmetry in men, while asymmetry in women is more associated with parietotemporal regions involved in language functions.

Our results suggest that sex-related differences in MPF persist after accounting for the linear component of age on myelination. A significant age effect, age-by-sex interaction, and negative linear correlation were found for absolute volumes of all compartments but for very few global and regional MPF measurements. Moreover, as in the work of Inano et al. [11], we found both negative and positive correlations of myelin-specific measurements with age. Negative correlations between regional measurements of MPF and age were observed mainly in men, while positive correlations were found mainly in women. A more detailed analysis with a quadratic model fit allowed us to estimate the peak age of maximum myelination for most regional MPF measurements, and it was older in women (46.4 ± 8.9) than in men (42.9 ± 5.5) by 3.5 years on average. The greatest shift towards older peak age in women was found for allocortex, deep GM, medial and occipital juxtacortical WM. Among all investigated regions, only projection WM pathways showed older peak age in males compared to females.

Numerous studies showed a significant effect of age on myelination [9,11,12,20,25,45,55,66,67]. Generally, linear positive correlations tend to be found in childhood and adolescence [55], while linear negative correlations tend to be found at ages over 60 years [20,66]. Studies with a wider range of participant ages from childhood to old age showed a better fit of myelin-specific measurements to the quadratic (inverted U-shaped) model than to the linear model [19,68,69,70]. Arshad et al. [69] investigated the relationship of DTI and MWF parameters with age within two models—linear and quadratic (inverted-U). It was found that FA and RD correlates with age only within a linear model, while MWF correlates with age within an inverted-U (quadratic) model, which better describes age-myelin association in the wide range of ages [68,69]. Similar results were reported by Faizy et al. [19]. Within the linear model, studies with samples of adults from young to old often show clear negative correlations only in a limited number of brain structures [25,67] or both negative and positive correlations [11]. In agreement with our results, Westley et al. [68] showed that within a quadratic model on a large sample and across a wide age range (8–85 years), DTI indices stabilized in the early fourth decade in all regions tested and then slowly declined. Tract-specific analyses for FA and RD showed the maximum values in the late third or early fourth decade in all tracts. The results of the present study also estimate the mean age of peak myelination as 43.5 ± 5.3 years. Our recent longitudinal study [45] with MRI scanning of the same subjects 7 years apart (mean age 44 years at first scanning and 51 years at second scanning) also showed a significant 5% decline in MPF with age.

Few of the above studies have examined both sex- and age-related differences in myelin-specific MRI parameters and were usually limited to a linear model [11,20,25]. In the age range 24.9–84.8 years, Inano et al. [11] found significant interaction between age and sex. The study by Hsu et al. [25] on participants from 30 to 80 years old showed that females have a significantly higher FA decrease than males in the right deep temporal region and the left side anterior limb of the IC. Brenner et al. [20] reported a significant age-by-sex interaction in the FX and cingulate cingulum but not in the hippocampal cingulum in older adults; MWF decreased with age in women but not men. In contrast, our results rather suggest an earlier and more prominent decrease in myelin density in men than in women, although we examined younger study participants. Inclusion of older participants in the sample would have helped to draw more reliable conclusions.

We found an age-independent significant effect of BMI on brain myelination: for both WM and mixed WM–GM, MFF values, which correlate with myelin content and peak age of myelination, were greater at higher BMI. These associations were evident regardless of sex, and the same trend was found in both men and women. Our results were somewhat unexpected, since most DTI (reviewed by Okudzhava et al. [29]) and MWF [30,31] studies found a weak but significant negative correlation between BMI and myelin. Myelin reduction in obese subjects is usually explained by such homeostatic disruptions as oxidative stress, neuroinflammation, and blood–brain barrier disruption [71,72]. Far fewer studies showed positive correlations between myelin and BMI or no correlation [29]. The discrepancy may likely be explained by significant differences in the metabolism of overweight and obese subjects and the different proportions of these subjects in the cohorts. It was shown that overweight individuals, unlike obese subjects, had a significantly higher mental health score [73] and lower mortality risk [74] compared with normal-weight individuals. Fatty acids, lipids, and cholesterol are critical building blocks for myelin synthesis [75]; therefore, an increase in the concentration of these components in overweight individuals, which does not lead to pathological changes in metabolism, may provide some advantages in myelin production in contrast to obese subjects. Undoubtedly, the positive association between BMI and myelination that we obtained should be interpreted with caution and tested on a larger sample and range of ages and BMI. Although this study is focused on the healthy population, it has certain clinical implications. Demyelination is commonly observed in numerous neurological conditions where it can be either a primary pathological substrate (for example, in MS) or a sequela of damage to axons, neurons, or oligodendroglia caused by neurodegenerative, traumatic, ischemic, toxic, infectious, and other injuries. The development of new therapies targeted at myelin repair drives the urgent need for a non-invasive biomarker of demyelination and remyelination that could be used as a surrogate endpoint in clinical trials. MPF provides an extensively validated, sensitive, and specific quantitative myelin biomarker, which can be measured with high acquisition speed and precision in clinical settings [43,44,45,51,52]. While the majority of clinical studies involving MPF were focused on MS (comprehensively reviewed in [37]), there are indications of potential clinical utility of MPF mapping in other neurological and psychiatric conditions, including neurodegenerative diseases [76,77,78,79], traumatic brain injury [50], brain tumors [80,81,82,83], and schizophrenia [44,84]. Recent animal studies demonstrated that MPF mapping may serve as a useful tool for monitoring of brain recovery after stroke [47,48]. The results of this study suggest that regardless of the clinical focus, age, sex, and, possibly, BMI, should be included as primary biological variables in the design of brain myelination studies in adults involving MPF mapping. Particularly, the quantitative detection of myelination abnormalities should be based on normative values in an appropriate age category with consideration of sex. Physiological interhemispheric asymmetry should be taken into account when appropriate, particularly in situations where MPF in unilateral lesions is compared to the contralateral hemisphere. In the studies involving a broad age range, it is imperative to include age as a covariate in both cross-sectional and longitudinal designs.

In conclusion, our study using a myelin-specific MPF mapping method clearly demonstrated sex-related global and regional differences, sex-dependent and sex-independent interhemispheric differences, and age-related sex differences in myelin density in adults. Higher brain myelination in males compared to females was found in global WM, most WM tracts, juxtacortical WM regions, and putamen. Most interhemispheric differences in MPF, especially for WM pathways and deep GM, are common to men and women. Sex-specific interhemispheric differences are mainly related to juxtacortical WM, and men are more likely to show left-sided asymmetry, while women are more likely to show right-sided asymmetry. Most investigated regions, including deep GM, juxtacortical WM, and WM tracts, except for projection tracts, showed a later peak age of myelination in women compared to men, with a difference of 3.5 years on average. The estimated peak age of maximum myelination in the female brain is on average 3.5 years later than in the male brain (46.4 ± 8.9 vs. 42.9 ± 5.5 years). BMI was associated with higher MPF and a later peak age of myelination independent of age and sex. The results of our current and previous findings [45,55] confirm the capability of fast MPF mapping to assess small sex-related differences and age-dependent changes in normal WM and GM myelination.

## Figures and Tables

**Table 1:** The demographic characteristics of participants of the study.

Parameter	Female	Male	Statistics
Sample size	23	19	-
Age, years ± SD	41.91 ± 10.95	42.97 ± 12.03	F(1, 39) = 0.03, *p* = 0.87
Age, median (min–max)	41 (20–61)	41 (23–67)	-
Body mass index (BMI)	24.98 ± 3.81	26.43 ± 4.98	F(1, 39) = 0.38, *p* = 0.54
Education, years ± SD	16.48 ± 2.19	16.11 ± 2.11	F(1, 39) = 0.29, *p* = 0.60

**Table 2:** Sex-dependent (Age × Sex) and sex-independent influence of age on MPF in global myelination.

Compartment, Parameter	Age			Age x Sex				
	ANCOVA, *p*	Total Sample		ANCOVA, *p*	Men		Women	
		Linear, Pearson’s r	Quadratic, Peak Age, Years		Linear, Pearson’s r	Quadratic, Peak Age, Years	Linear Pearson’s r	Quadratic, Peak Age, Years
Mixed WM–GM, MPF	0.049	n.s.	46.9 ***	n.s.	n.s.	45.5 ***	0.49 *	50.1 ***
WM, MPF	n.s.	n.s.	43.1 ***	n.s.	n.s.	43.2 ***	n.s.	45.4 ***
CSF, volume	n.s.	n.s.	-	0.04	−0.55 *	-	n.s.	-
GM, volume	0.008	−0.43 **	-	0.01	−0.73 **	-	n.s.	-
Mixed WM–GM, volume	0.02	−0.42 **	-	0.047	−0.72 **	-	n.s.	-
WM, volume	0.04	−0.33 *	-	0.02	−0.64 **	-	n.s.	-
Total brain volume	0.03	−0.37 *	-	0.002	−0.69 **	-	n.s.	-
CSF, percentage volume	0.0003	0.44 **	-	n.s.	0.71 **	-	n.s.	-
GM, percentage volume	n.s.	n.s.	-	n.s.	n.s.	-	n.s.	-
Mixed WM–GM, percentage volume	n.s.	n.s.	-	0.02	n.s.	-	−0.64 **	-
WM, percentage volume	n.s.	n.s.	-	n.s.	n.s.	-	n.s.	-
WM/GM volume	n.s.	n.s.	-	n.s.	n.s.	-	n.s.	-

Significant linear Pearson’s correlations: *—*p* < 0.05, **—*p* < 0.01, ***—*p* < 0.001. Significance of the best quadratic model fitting: ***—*p* < 0.001. Age × Sex shows statistical interaction between sex and age variables.

**Table 3:** Sex-dependent and sex-independent influence of age in the MPF of separate brain regions.

Group of Structures	Significant Quadratic Equations, N			Peak Age, Years ± SD		
	Total Sample	Men	Women	Total Sample	Men	Women
Frontal WM	14	14	11	41.2 ± 4.0	40.7 ± 1.8	42.2 ± 5.7
Parietal WM	7	7	3	42.7 ± 5.0	40.4 ± 3.3	41.5 ± 1.8
Occipital WM	10	10	8	43.4 ± 4.1	40.6 ± 6.2	45.0 ± 8.2
Temporal WM	6	6	0	44.2 ± 0.9	42.9 ± 0.3	-
Medial WM	8	8	6	42.9 ± 3.1	42.6 ± 3.0	50.2 ± 12.5 *
All juxtacortical WM	45	45	28	42.6 ± 3.8	41.1 + 3.3	44.6 + 8.3 *
Projection WM	28	27	26	46.8 ± 7.7	47.5 ± 7.8	44.6 ± 8.6 *
Association WM	12	12	12	44.0 ± 3.5	43.4 ± 2.6	47.4 ± 9.3 *
Commissural WM	12	12	8	41.7 ± 3.7	41.5 ± 3.2	45.0 ± 6.8
All WM pathways	52	51	44	45.0 + 6.5	45.1 + 6.5	45.7 + 8.5
Allocortex and deep GM	14	14	12	41.0 ± 2.4	39.8 ± 4.4	51.2 ± 8.8 ***
Brainstem	3	3	3	40.8 ± 9.0	43.0 ± 5.9	44.2 ± 3.7
All structures	117	116	90	43.5 ± 5.3	42.9 ± 5.5	46.4 ± 8.9 **

Significant difference in the peak age of myelination between men and women for adjacent groups of structures: *—*p* < 0.05, **—*p* < 0.01, ***—*p* < 0.001.

**Table 4:** Sex-dependent (BMI × Sex) and sex-independent influence of BMI on the MPF in global myelination within linear model.

Compartment, Parameter	BMI		BMI x Sex		
	Total Sample		ANCOVA, *p*	Men	Women
	ANCOVA, *p*	Partial Correlations, r		Partial Correlations, r	Partial Correlations, r
Mixed WM–GM, MPF	0.048	0.35 *	n.s.	n.s.	0.49 *
WM, MPF	0.049	0.34 *	n.s.	n.s.	n.s.
CSF, volume	n.s.	n.s.	n.s.	n.s.	n.s.
GM, volume	n.s.	n.s.	n.s.	n.s.	n.s.
Mixed WM–GM, volume	n.s.	n.s.	n.s.	n.s.	n.s.
WM, volume	n.s.	n.s.	n.s.	n.s.	n.s.
Total brain volume	n.s.	n.s.	n.s.	n.s.	n.s.
CSF, percentage volume	n.s.	n.s.	0.002	n.s.	−0.54 **
GM, percentage volume	n.s.	n.s.	n.s.	n.s.	n.s.
Mixed WM–GM, percentage volume	n.s.	n.s.	n.s.	n.s.	n.s.
WM, percentage volume	n.s.	n.s.	n.s.	n.s.	n.s.
WM/GM volume	n.s.	n.s.	n.s.	n.s.	n.s.

Significant age-controlled partial correlations: *—*p* < 0.05, **—*p* < 0.01. BMI × Sex shows statistical interaction between BMI and sex variables.

**Table 5:** Sex-dependent and sex-independent influence of BMI on the peak age of global myelination within quadratic model.

Compartment	BMI			BMI x Sex					
	Total Sample			Men			Women		
	Total	BMInorm	BMIex	Total	BMInorm	BMIex	Total	BMInorm	BMIex
Mixed WM–GM, peak age, years	46.9 *	44.9 *	47.5 *	45.5 *	42.8 *	47.3 *	50.1 *	48.7 *	50.1 *
WM, peak age, years	43.1 *	40.2 *	44.7 *	43.2 *	40.5 *	45.9 *	45.4 *	40.4 *	45.6 *

Significance of the best quadratic model fitting: *—*p* < 0.001.

## Data Availability

Data are unavailable due to privacy or ethical restrictions.

## Supplementary Materials

The following supporting information can be downloaded at: https://www.mdpi.com/article/10.3390/jcm13237065/s1, Figure S1: Example MPF maps, T1, T2, and T2-FLAIR images of male (46 years) and female (48 years) study participants; Table S1: Sex-dependent and sex-independent influence of age in the MPF of separate brain structures.
