# Supplementary material for: Global and Regional Sex-Related Differences, Asymmetry, and Peak Age of Brain Myelination in Healthy Adults
Source: J Clin Med. 2024 Nov 22;13(23):7065. doi: 10.3390/jcm13237065 (PMC11642669; doi:10.3390/jcm13237065)
Supplement: Supplementary file 1 [file jcm-13-07065-s001.zip › jcm-3276037 Supplementary Figure.pdf]

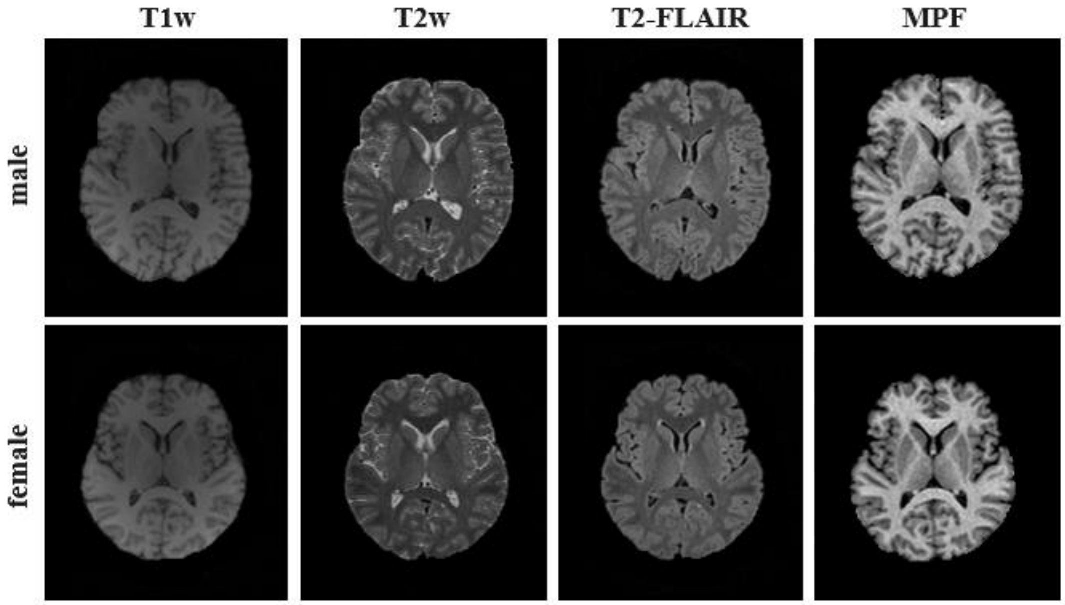

Supplementary Figure S1. Example MPF maps, T1, T2, and T2-FLAIR images of male (46 years) and female (48 years) study participants.
