# Supplementary material for: Global and Regional Sex-Related Differences, Asymmetry, and Peak Age of Brain Myelination in Healthy Adults
Source: J Clin Med. 2024 Nov 22;13(23):7065. doi: 10.3390/jcm13237065 (PMC11642669; doi:10.3390/jcm13237065)
Supplement: Supplementary file 1 [file jcm-13-07065-s001.zip › jcm-3276037 Supplementary Table.pdf]

Supplementary Table S1. Sex-dependent and sex-independent influence of age in the MPF of separate brain structures.

Area	Brain structures	Age			Age x Gender				
			Total sample			Men		Women	
		ANCOVA, p (L/R)	Linear, Pearson's r (L/R)	Quadratic, peak age, years (L/R)	ANCOVA, p (L/R)	Linear, Pearson's r (L/R)	Quadratic, peak age, years (L/R)	Linear, Pearson's r (L/R)	Quadratic, peak age, years (L/R)
Frontal WM	Lateral fronto-orbital	0.02 / n.s.	-0.31* / n.s.	38.1*/ 39.7*	0.01/0.03	-0.55*/ -0.52*	36.9*/ 40.6*	n.s.	43.5*/40.1*
	Middle fronto-orbital	0.03 / n.s.	-0.35* / n.s.	37.8* / 39.6*	0.01/0.03	n.s.	38.8* / 40.0*	n.s.	36.8*/40.6*
	Rectus	n.s. / 0.04	n.s.	39.9* / 39.1*	n.s.	n.s.	41.4* / 41.0*	n.s.	38.8* / 32.4*
	Superior frontal	n.s.	n.s.	41.2* / 40.5*	n.s.	n.s.	41.9* / 41.4*	n.s.	42.3* / 40.6*
	Inferior frontal	n.s.	n.s.	41.4* / 40.6*	n.s. / 0.001	n.s. / -0.62**	40.2* / 39.0*	n.s.	51.0*/ n.s.
	Middle frontal	n.s.	n.s.	37.4* / 43.4*	n.s.	n.s.	39.8* /43.6*	n.s.	48.3* / 49.8*
	Precentral	n.s.	n.s.	53.4* / 44.6*	n.s.	n.s.	43.9* / 41.3*	n.s.	n.s.
Parietal WM	Angular	n.s.	n.s.	41.2* / 40.0*	n.s.	n.s. / -0.50*	40.5* / 37.5*	n.s.	n.s.
	Postcentral	n.s.	n.s.	49.2* / 50.2*	n.s.	n.s.	43.8* / 44.5*	n.s.	n.s.
	Superior parietal	n.s.	n.s.	41.4* / 39.3*	n.s.	n.s.	41.4* / 39.9*	n.s.	43.1* / 39.6*
	Supramarginal	n.s.	n.s.	n.s. / 37.5*	n.s.	n.s.	n.s. / 35.3*	n.s.	n.s. / 41.9*
Occipital WM	Cuneus	n.s.	n.s.	52.2* / 46.4*	n.s. / 0.04	n.s. / n.s.	45.9* /27.2*	n.s.	63.0* / n.s.
	Lingual	n.s.	n.s.	40.9* / 38.4*	n.s.	n.s.	37.0* / 36.9*	n.s.	41.9* / 40.1*
	Superior occipital	n.s.	n.s.	41.1* / 41.5*	n.s.	n.s.	42.9* / 42.0*	n.s.	n.s. /38.0
	Inferior occipital	n.s.	n.s.	47.2* / 41.1*	n.s. / 0.03	n.s. / -0.46*	49.7* / 38.1*	n.s.	41.2* / 47.2*
	Middle occipital	n.s.	n.s.	44.5* / 41.1*	n.s.	n.s.	43.1* / 42.9*	n.s.	49.0* / 39.3*
Temporal WM	Superior temporal	n.s.	n.s.	45.2* / 43.0*	n.s.	n.s.	43.1* / 42.7***	n.s.	n.s.
	Inferior temporal	n.s.	n.s.	44.6* / 45.0*	n.s.	n.s.	43.1*/ 43.1*	n.s.	n.s.

	Middle temporal	n.s.	n.s.	44.0* / 43.4*	n.s.	n.s.	42.5* / 42.8*	n.s.	n.s.
Medial WM	Pre-cuneus	n.s.	n.s.	40.0* / 42.2*	n.s.	n.s.	40.2* / 47.7*	n.s.	45.8* / 37.5*
	Cingulum (cingulate)	n.s.	n.s.	43.8* / 37.8*	n.s.	n.s. / -0.47*	43.5* / 37.7*	n.s.	69.5* / 37.2*
	Cingulum (hippocampus)	n.s.	n.s.	45.1* / 44.5*	n.s.	n.s.	43.9* / 42.5*	n.s.	55.7* / n.s.
	Fusiform	n.s.	n.s.	42.2*/ 47.9*	n.s.	n.s.	41.1* / 44.2*	n.s. / 0.48*	55.4* / n.s.
Projection WM pathways	CST	n.s.	0.30* / n.s.	57.1* / 55.9*	n.s.	0.46* / n.s.	57.8*/ 58.7*	n.s.	47.1*/46.1*
	Anterior CR	n.s.	n.s.	38.6* / 38.7*	n.s.	n.s.	41.2* / 41.0*	n.s.	33.6* / 33.1*
	Superior CR	n.s.	n.s.	41.8* / 40.0*	n.s.	n.s.	43.3* / 41.4*	n.s.	40.5* / 39.1*
	Posterior CR	n.s.	n.s.	39.3* / 39.3*	n.s.	n.s.	40.1* / 38.0*	n.s.	39.6* /37.1*
	Anterior limb IC	n.s.	n.s.	42.0* / 43.3*	0.02 / 0.02	n.s.	40.9* / 42.2*	n.s.	53.2* / 61.1*
	Posterior limb IC	n.s.	n.s.	45.7* / 44.2*	n.s.	n.s.	45.8* / 44.6*	n.s.	46.4* / 46.6*
	Retrolenticular IC	n.s.	n.s.	45.7* / 46.2*	n.s.	n.s.	41.4* / 44.4*	n.s.	n.s.
	Inferior CP	n.s. / 0.04	n.s. / 0.36*	47.4* / 65.0*	n.s.	n.s.	47.9* / 68.1*	n.s.	47.8* / 63.7*
	Middle CP	0.02 / 0.02	0.42** / 0.41**	57.4* / 56.3*	n.s.	0.61** / 0.47*	57.8* / 56.5*	n.s. / 0.43	47.8* / 52.3*
	Superior CP	n.s.	n.s.	40.0* / 39.8*	n.s.	n.s.	49.1* / 43.7*	n.s.	32.6* / 33.3*
	Cerebral peduncles	n.s.	n.s.	48.1* / 48.4*	n.s.	n.s.	49.8* / 48.9*	n.s.	44.0* / 45.5*
	Posterior thal. rad.	0.03 / 0.008	n.s. / -0.32*	39.3* /37.0*	n.s.	n.s.	40.3* /39.8*	n.s.	38.1* / 31.6*
	Medial lemniscus	n.s.	n.s.	47.6* / 49.8*	n.s.	n.s.	48.4* / 52.1*	n.s.	46.2* / 43.3*
	Pontine crossing tract	0.02 / 0.001	0.38* / 0.51***	58.0* / 58.2*	n.s.	n.s.	n.s. / 60.1*	0.42*/ n.s.	54.4* / 54.6*
Commissural WM pathways	Genu CC	n.s. / 0.01	n.s. /-0.36*	38.9* / 37.0*	n.s.	n.s. / -0.57*	40.6* / 38.1*	n.s.	n.s.
	Body CC	n.s.	n.s.	42.1* / 39.3*	n.s.	n.s. / -0.55*	45.0* / 37.6*	n.s.	43.0* / 43.5*
	Splenium CC	n.s.	n.s.	46.0* / 45.1*	n.s.	n.s.	45.0* / 44.4*	0.43* / n.s.	58.4* / 47.8*
	Tapetum	n.s.	n.s.	40.7* / 36.9*	n.s.	n.s.	42.2* / 36.8*	n.s.	38.9* / 37.0*
	FX column and body	n.s.	n.s.	42.1* / 39.2*	n.s.	n.s. / -0.61**	41.8* /37.8*	n.s.	49.6* / 41.9*

	FX stria terminalis	n.s.	n.s.	45.6* / 47.8*	n.s.	n.s.	44.9* / 43.4*	0.46* / n.s.	n.s.
Association WM pathways	Sagittal stratum	n.s.	n.s.	41.2* / 39.7*	n.s.	n.s.	41.1* / 40.4*	n.s.	51.1* / n.s.
	IFOF	n.s.	n.s.	46.6* / 46.2*	0.04 / 0.02	n.s.	43.7* / 43.4*	0.41* / 0.43*	65.2* / 60.9*
	SFOF	n.s.	n.s.	38.8* / 41.1*	n.s.	n.s.	40.5* / 42.4*	n.s.	37.4* / 40.4*
	Uncinate fasciculus	n.s.	n.s. / 0.34*	45.1* / 49.2*	n.s.	n.s.	45.8* / 48.4*	n.s. / 0.43*	43.6* / 48.2*
	SL fasciculus	n.s.	n.s.	47.6* / 40.6*	n.s.	n.s.	46.5* / 40.6 *	n.s.	46.6* / 41.4*
	External capsule	n.s.	n.s.	46.1* / 45.3*	n.s.	n.s.	44.5* / 43.3*	n.s.	56.75* / n.s.
Deep GM and allocortex	Amygdala	n.s.	n.s.	42.6* / 39.9*	n.s.	n.s.	29.9* / 39.3*	n.s.	47.3* / 40.1*
	Entorhinal area	n.s.	n.s.	36.6* / 38.5*	n.s.	n.s.	41.9* / 46.0*	n.s.	n.s.
	Caudate nucleus	0.006 / 0.0008	-0.38* / -0.44**	38.8* / 38.0*	0.005 / 0.0005	-0.64** / -0.73***	38.1* / 36.4*	n.s.	42.8* / 43.8*
	Globus pallidus	n.s.	n.s.	43.6* / 43.3*	n.s.	n.s.	42.3* / 43.3*	n.s.	50.4* / 50.4*
	Putamen	n.s.	n.s.	43.8* / 43.2*	n.s.	n.s.	43.3* / 42.7*	n.s.	47.8* / 52.6*
	Hippocampus	n.s.	n.s.	40.3* / 41.0*	0.01 / 0.009	-0.55* / -0.50*	32.8* / 37.4*	n.s.	49.4* / 61.4*
	Thalamus	n.s.	n.s.	42.5* / 42.1*	0.04 / 0.02	n.s.	41.7* / 41.3*	n.s.	56.2* / 72.1*
Brainstem	Medulla	n.s.	n.s.	41.5*	n.s.	n.s.	39.4*	n.s.	47.7*
	Midbrain	n.s.	n.s.	31.5*	n.s.	n.s.	43.0*	n.s.	40.3*
	Pons	n.s.	n.s.	49.5*	n.s.	n.s.	51.0*	n.s.	44.6*

Significant linear Pearson's correlations: * - p<0.05, ** - p<0.01, *** - p<0.001. Significance of the best quadratic model fitting, F-criteria: * - p<0.001. Abbreviations: n.s. – non-significant, L – left hemisphere, R – right hemisphere.
